# MVIAeval: a web tool for comprehensively evaluating the performance of a new missing value imputation algorithm

**DOI:** 10.1186/s12859-016-1429-3

**Published:** 2017-01-13

**Authors:** Wei-Sheng Wu, Meng-Jhun Jhou

**Affiliations:** Department of Electrical Engineering, National Cheng Kung University, Tainan, Taiwan

**Keywords:** Web tool, Missing value imputation, Microarray data, Performance index, Performance comparison, Algorithm

## Abstract

**Background:**

Missing value imputation is important for microarray data analyses because microarray data with missing values would significantly degrade the performance of the downstream analyses. Although many microarray missing value imputation algorithms have been developed, an objective and comprehensive performance comparison framework is still lacking. To solve this problem, we previously proposed a framework which can perform a comprehensive performance comparison of different existing algorithms. Also the performance of a new algorithm can be evaluated by our performance comparison framework. However, constructing our framework is not an easy task for the interested researchers. To save researchers’ time and efforts, here we present an easy-to-use web tool named MVIAeval (Missing Value Imputation Algorithm evaluator) which implements our performance comparison framework.

**Results:**

MVIAeval provides a user-friendly interface allowing users to upload the R code of their new algorithm and select (i) the test datasets among 20 benchmark microarray (time series and non-time series) datasets, (ii) the compared algorithms among 12 existing algorithms, (iii) the performance indices from three existing ones, (iv) the comprehensive performance scores from two possible choices, and (v) the number of simulation runs. The comprehensive performance comparison results are then generated and shown as both figures and tables.

**Conclusions:**

MVIAeval is a useful tool for researchers to easily conduct a comprehensive and objective performance evaluation of their newly developed missing value imputation algorithm for microarray data or any data which can be represented as a matrix form (e.g. NGS data or proteomics data). Thus, MVIAeval will greatly expedite the progress in the research of missing value imputation algorithms.

## Background

Microarray technology is one of the most powerful high-throughput tools in biomedical and biological research. It has been successfully applied to various studies such as cancer classification [1], drug discovery [2], stress response [3, 4], and cell cycle regulation [5, 6]. Microarray data contain missing values due to various technological limitations such as poor hybridization, spotting problems, insufficient resolution, and fabrication errors. Unfortunately, the missing values in microarray data would significantly degrade the performance of downstream analyses such as gene clustering and identification of differentially expressed genes [7–9]. Therefore, missing value imputation has become an important pre-processing step in microarray data analyses.

One way to deal with the missing values is to repeat the experiments but it is expensive and time consuming. Another way is to discard the genes with missing values but this loses valuable information. Filling missing values with zeros or with the row average is a simple imputation strategy, but it is far from optimal. Therefore, many advanced algorithms have been developed to impute the missing values in microarray data [10–12]. The existing algorithms can be divided into four categories [11]: global approach, local approach, hybrid approach and knowledge-assisted approach. Global approach algorithms include SVD [13] and BPCA [14]. Local approach algorithms include KNN [13], SKNN [15], IKNN [16], LS [17], LLS [18], SLLS [19], ILLS [20], Shrinkage LLS [21] and so on. Hybrid approach algorithms include LinCmb [22] and RMI [23]. Knowledge-assisted approach algorithms include GOimpute [24], POCSimpute [25] and HAIimpute [26].

In order to know which algorithm performs best among the dozens of existing ones, an objective and comprehensive performance comparison framework is urgently needed. To meet the need, we previously developed a performance comparison framework [12] which provides 13 testing microarray datasets, three types of performance indices, 9 existing algorithms, and 110 runs of simulation. We found that no single algorithm can perform best for all types of microarray data. The best algorithms are different for different microarray data types (time series and non-time series) and different performance indices, showing the usefulness of our framework for conducting a comprehensive performance comparison [12].

Actually, the most important value of our framework is to give an objective and comprehensive performance evaluation of a new algorithm. Using our framework, bioinformaticians who design new algorithms can easily know their algorithms’ performance and then refine their algorithms if needed. However, constructing our framework is not an easy task for the interested bioinformaticians. It involves collecting and processing many microarray raw data from the public domain and using programming languages to implement many existing algorithms and three performance indices. In order to save bioinformaticians’ efforts and time, we present an easy-to-use web tool named MVIAeval (Missing Value Imputation Algorithm evaluator) which implements our performance comparison framework.

## Implementation

### Twenty benchmark microarray datasets and twelve existing algorithms used for performance comparison

In MVIAeval, we collected 20 benchmark microarray datasets [27–46] of different species and different types (see Table 1 for details). In addition, we implemented 12 existing algorithms including two global approach algorithms and 10 local approach algorithms (see Table 2 for details). Do note that we did not include hybrid approach algorithms and knowledge-assisted algorithms because they either are difficult to implement or need extra information from outside data sources which are not always available.

**Table 1:** The 20 benchmark microarray datasets of different types and different species

GEO Dataset	Size	Type	Organism	Title
GDS3323 [27]	45101x6	Non-time series	Mus musculus	Na+/H+ exchanger 3 deficiency effect on the colon
GDS3215 [28]	12625x6	Non-time series	*Homo sapiens*	13-cis retinoic acid effect on SEB-1 sebocyte cell line
GDS3485 [29]	45011x6	Non-time series	*Mus musculus*	Zinc transporter SLC39A13 deficiency effect on chondrocytes
GDS3476 [30]	45011x6	Non-time series	*Mus musculus*	NF-E2-related factor 2 Nrf2 activation effect on the liver
GDS3197 [31]	45101x6	Non-time series	*Mus musculus*	Transcriptional coactivator PGC-1beta hypomorphic mutation effect on the liver
GDS3149 [32]	45101x6	Non-time series	*Mus musculus*	Suppressor of cytokine signaling 3 deficiency effect on the regenerating liver
GDS2107 [33]	15923x6	Non-time series	*Rattus* * norvegicus*	Long-term ethanol consumption effect on pancreas
GDS3464 [34]	15617x6	Non-time series	*Danio rerio*	SPT5 mutant embryos
GDS3426 [35]	23015x6	Non-time series	*Staphylococcus* * epidermidis*	Staphylococcus epidermidis SarZ mutant
GDS3421 [36]	10208x6	Non-time series	*Escherichia* * coli*	Frag1 cells response to ionic and non-ionic hyperosmotic stress
GDS3360 [37]	22575x8	Time series	*Homo sapiens*	Chlamydia pneumoniae infection effect on HL epithelial cells: time course
GDS2863 [38]	31099x6	Time series	Rattus norvegicus	Tienilic acid effect on the liver: time course
GDS5057 [39]	34760x8	Time series	*Mus musculus*	Mepenzolate bromide effect on lung: time course
GDS5055 [40]	45307x10	Time series	*Mus musculus*	Histone demethylase KDM1A deficiency effect on 3 T3-L1 preadipocytes: time course
GDS3428 [41]	22283x9	Time series	*Homo sapiens*	Immature dendritic cell response to butanol fraction of Echinacea purpurea: time course
GDS4484 [42]	45101x8	Time series	*Mus musculus*	Cerebellar neuronal cell response to thyroid hormone: time course
GDS3785 [43]	17589x8	Time series	Homo sapiens	Osteoarthritic chondrocytes and healthy mesenchymal stem cell during chondrogenic differentiation: time course
GDS3930 [44]	8799x9	Time series	*Rattus* * norvegicus*	Bone morphogenic protein effect on cultured sympathetic neurons: time course
GDS4321 [45]	10208x8	Time series	*Escherichia* * coli*	Escherichia coli O157:H7 response to cinnamaldehyde: time course
GDS3032 [46]	22277x8	Time series	Homo sapiens	Quercetin effect on intestinal cell differentiation in vitro: time course

**Table 2:** The 12 existing algorithms implemented in MVIAeval

Algorithm	Year of Publication	Category	Reference
SVD	2001	Global	[13]
BPCA	2003	Global	[14]
KNN	2001	Local	[13]
SKNN	2004	Local	[15]
IKNN	2007	Local	[16]
LS	2004	Local	[17]
LLS	2005	Local	[18]
ILLS	2006	Local	[19]
SLLS	2008	Local	[20]
Shrinkage LLS	2013	Local	[21]
Shrinkage SLLS	2013	Local	[21]
Shrinkage ILLS	2013	Local	[21]

### Three existing performance indices used for performance evaluation

**Fig. 1:**
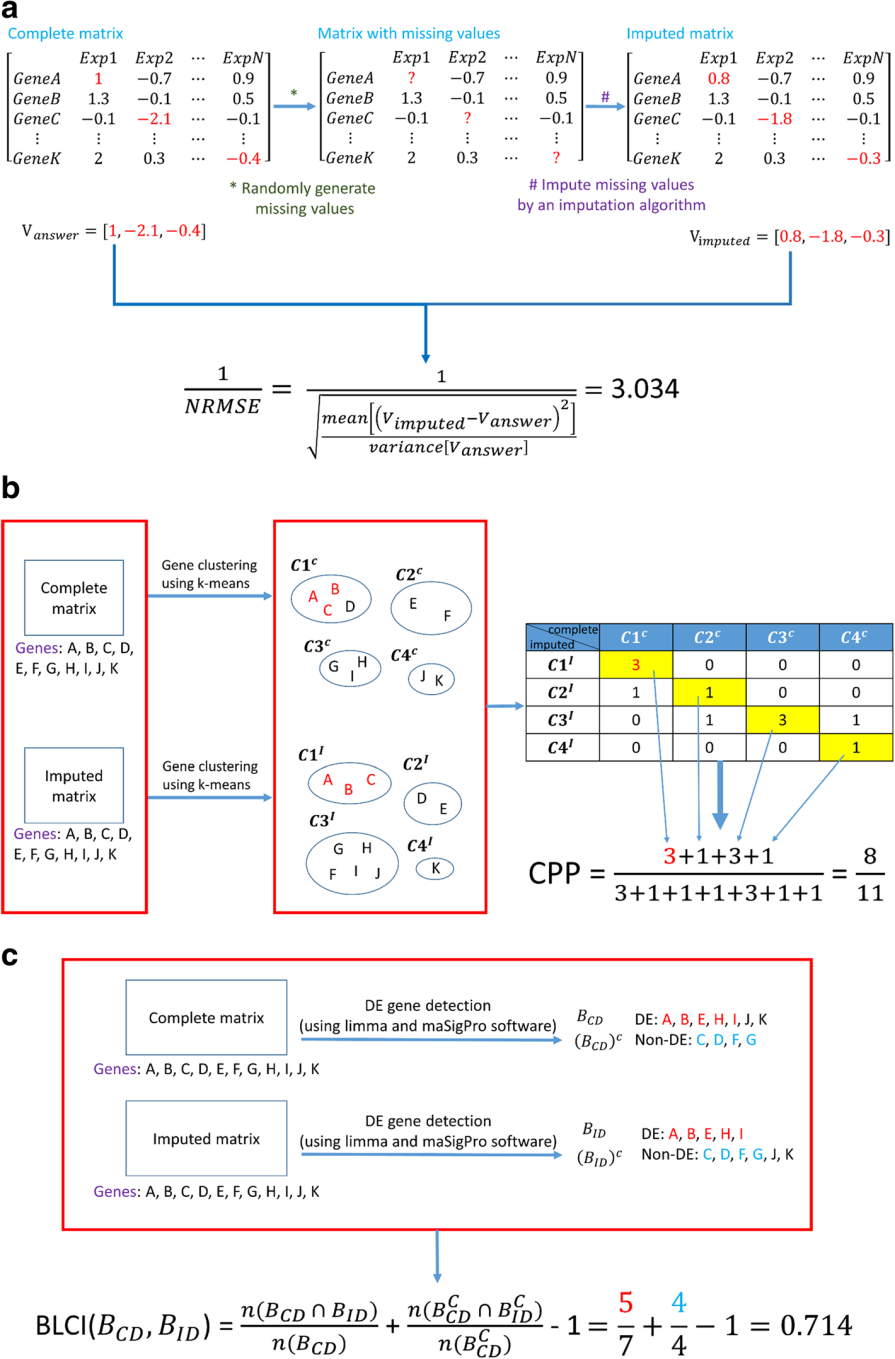
Three performance indices implemented in MVIAeval. MVIAeval implements three performance indices, which are **a** 1/NRMSE, **b** CPP and **c** BLCI. Here we provide an example to show how the scores of these three performance indices are calculated

In MVIAeval, we used three existing performance indices for performance evaluation. First, the inverse of the normalized root mean square error (1/NRMSE) [13] is used to measure the numerical similarity between the imputed matrix (generated by an imputation algorithm) and the original complete matrix. Therefore, the higher the 1/NRMSE value is, the better the performance of an imputation algorithm is. Second, the cluster pair proportion (CPP) [47] is used to measure the similarity of the gene clustering results of the imputed matrix and the complete matrix. High CPP value means that the imputed matrix (generated by an imputation algorithm) has very similar gene clustering results as the complete matrix does. Therefore, the higher the CPP value is, the better the performance of an imputation algorithm is. Third, the biomarker list concordance index (BLCI) [7] is used to measure the similarity of the differentially expressed genes identification results of the imputed matrix and the complete matrix. High BLCI value means that differentially expressed genes identified using the imputed matrix (generated by an imputation algorithm) are very similar to those identified using the complete matrix. Therefore, the higher the BLCI value is, the better the performance of an imputation algorithm is. In summary, 1/NRMSE measures the numerical similarity, while CPP and BLCI measure the similarity of downstream analysis results (gene clustering and differentially expressed genes identification) of the imputed matrix and the complete matrix. Fig. 1 shows how the scores of these three performance indices are calculated.

### Evaluating the performance of an algorithm for a benchmark microarray data matrix using a specific performance index

**Fig. 2:**
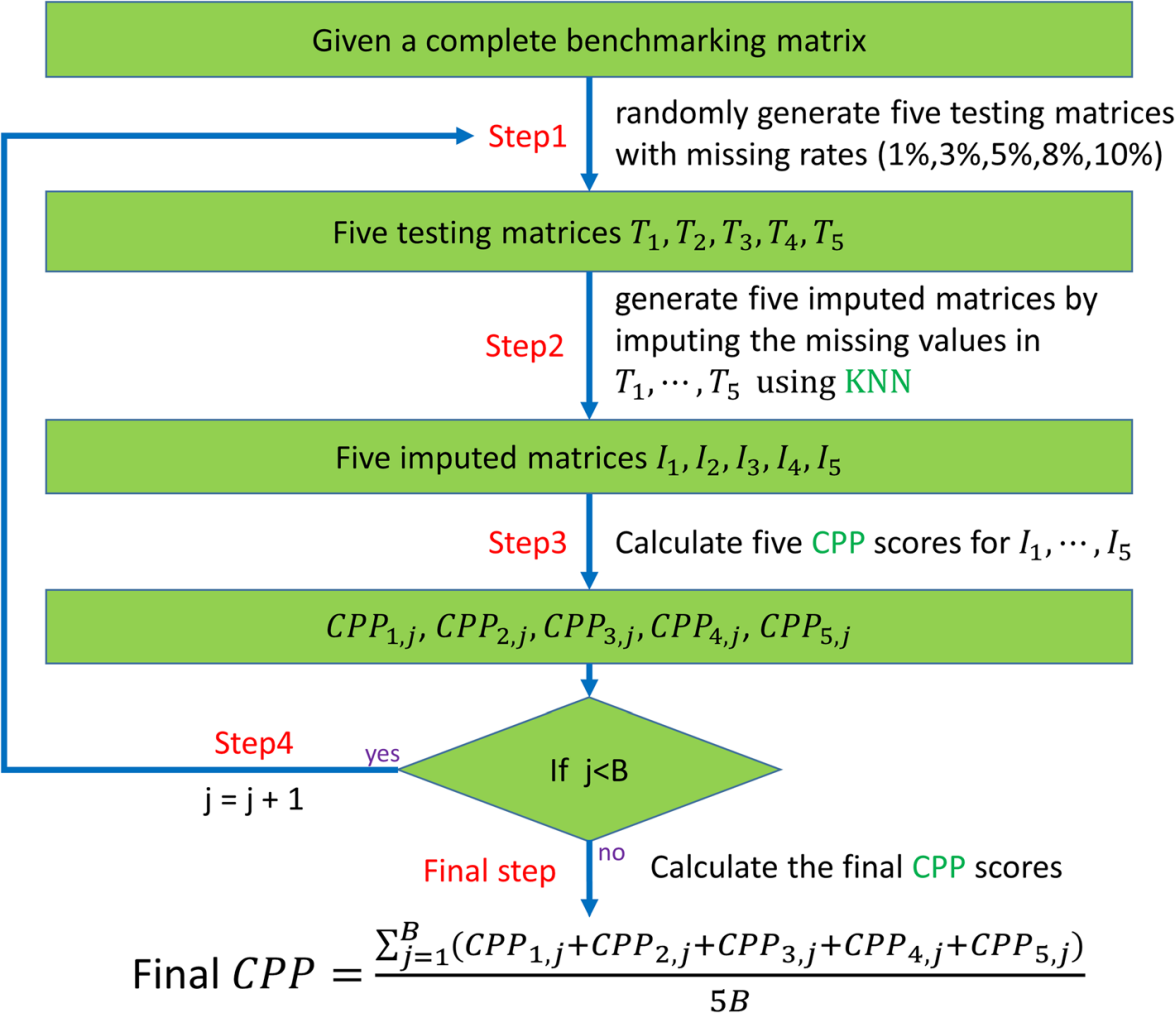
The simulation procedure for evaluating the performance of an algorithm. The simulation procedure for evaluating the performance of an imputation algorithm (e.g. KNN) for a given complete benchmark microarray data matrix using a performance index (e.g. CPP) is divided into four steps

The simulation procedure for evaluating the performance of an imputation algorithm (e.g. KNN) for a given complete benchmark microarray data matrix using a performance index (e.g. CPP) is divided into four steps. **Step 1:** generate five testing matrices having missing values (generated as missing completely at random) with different percentages (1%, 3%, 5%, 8% and 10%) from the complete matrix. **Step 2:** generate five imputed matrices by imputing the missing values in the five testing matrices using KNN. **Step 3**: calculate five CPP scores using the complete matrix and five imputed matrices. **Step 4**: repeat Steps 1–3 for *B* times, where *B* is the number of simulation runs per missing percentage. Then the final CPP score of KNN for the given benchmark microarray data matrix is defined as the average of the 5*B CPP scores. Fig. 2 illustrates the whole simulation procedure.

### Two existing comprehensive performance scores

In MVIAeval, we implemented two existing comprehensive performance scores [48, 49] to provide the overall performance comparison results for the selected benchmark microarray datasets and performance indices. The first one, termed the overall ranking score (ORS), is defined as the sum of the rankings of an algorithm for the selected performance indices and benchmark microarray datasets [48, 49]. The ranking of an algorithm for a specific performance index and a specific benchmark microarray dataset is *d* if its performance ranks *#d* among all the compared algorithms. For instance, the best algorithm has ranking 1. Therefore, small ORS indicates that an algorithm has good overall performance.

The other comprehensive performance score, termed the overall normalized score (ONS), is calculated by the sum of the normalized scores for the benchmark microarray datasets and performance indices [48, 49]. The ONS of the algorithm *k* is calculated like the following:

$$ ONS(k)={\displaystyle \sum_{i=1}^I}{\displaystyle \sum_{j=1}^{\mathrm{J}}}{N}_{ij}(k)={\displaystyle \sum_{i=1}^I}{\displaystyle \sum_{j=1}^{\mathrm{J}}}\left(\frac{S_{ij}(k)}{ \max \left({S}_{ij}(1),{S}_{ij}(2),\dots, {S}_{ij}(m)\right)}\right) $$

where *S*_*ij*_(*k*) and *N*_*ij*_(*k*) is the original score and the normalized score of the algorithm *k* for the selected performance index *i* and benchmark microarray dataset *j*, respectively; *I* is the number of the selected indices; *J* is the number of the selected benchmark microarray datasets and *m* is the number of the algorithms being compared. Note that 0 ≤ *N*_*ij*_(*k*) ≤ 1 and *N*_*ij*_(*k*) = 1 when the algorithm *k* performs best for the selected performance index *i* and benchmark microarray dataset *j* (i.e. S_*ij*_(*k*) = max(*S*_*ij*_(1), *S*_*ij*_(2), …, *S*_*ij*_(*m*))). Therefore, large ONS indicates that an algorithm has good overall performance.

## Results and discussion

### Usage

**Fig. 3:**
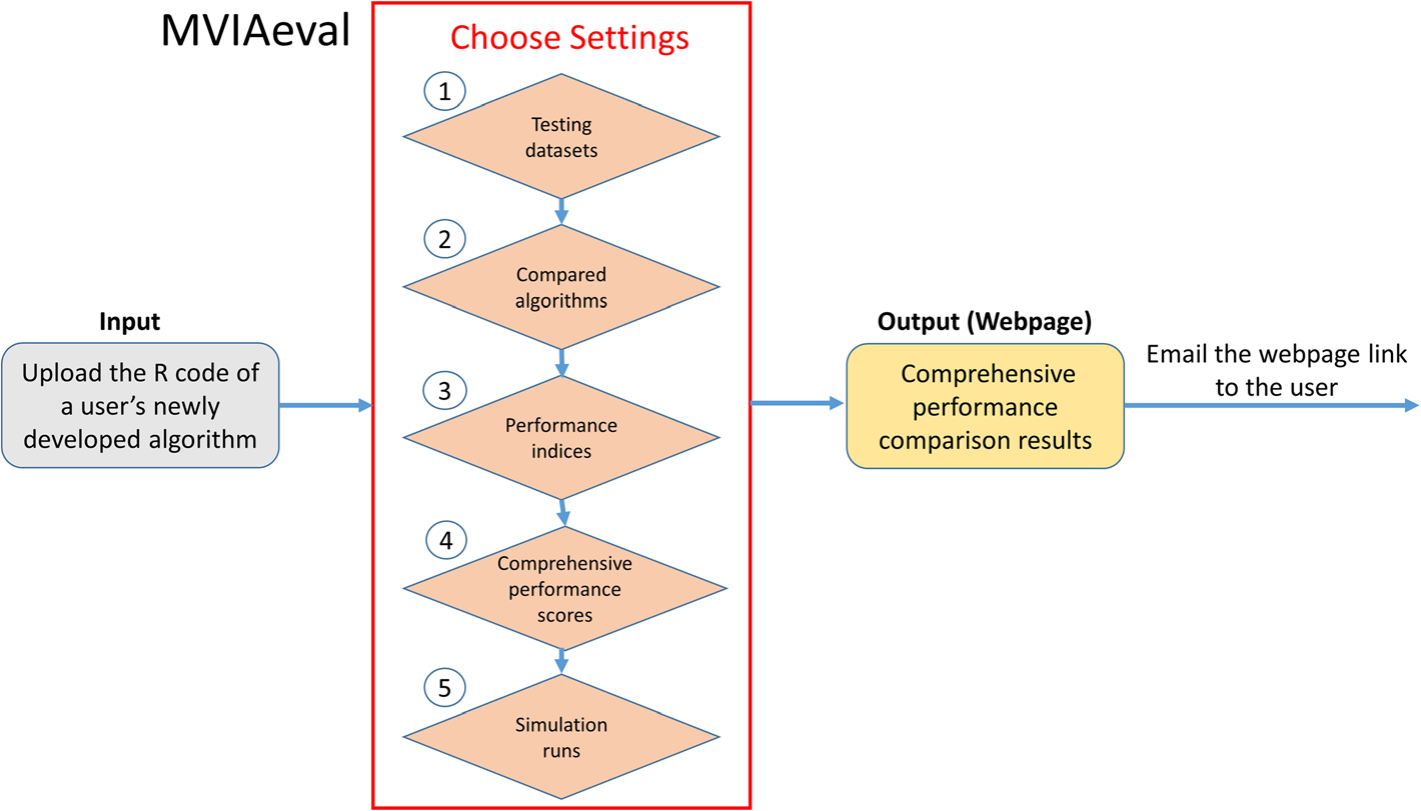
The flowchart of MVIAeval. The flowchart shows how MVIAeval conducts a comprehensive performance comparison for a new algorithm

Figure 3 illustrates the usage of MVIAeval. The easy-to-use web interface allows users to upload the R code of their newly developed algorithm. Subsequently five types of settings of MVIAeval need to be set. First, the test datasets have to be chosen from 20 benchmark microarray datasets. The collected benchmark datasets consist of two types of data: 10 non-time series data and 10 time series data. Second, the compared algorithms have to be chosen from 12 existing algorithms. The collected existing algorithms consist of two global approach algorithms and 10 local approach algorithms. Third, the performance indices have to be chosen from three existing ones (1/NRMSE, CPP and BLCI). Fourth, the comprehensive performance scores have to be chosen from two existing ones (ORS and ONS). Fifth, the number of simulation runs have to be specified. The larger the number of simulation runs is, the more accurate the comprehensive performance comparison result is. But be cautious that the simulation time increases linearly with the number of simulation runs. After submission, a comprehensive performance comparison between the user’s algorithm and the selected existing algorithms is executed by MVIAeval using the selected benchmark datasets and performance indices. Then a webpage of the comprehensive performance comparison results is generated and the webpage link is sent to the users by e-mails.

### A case study

**Fig. 4:**
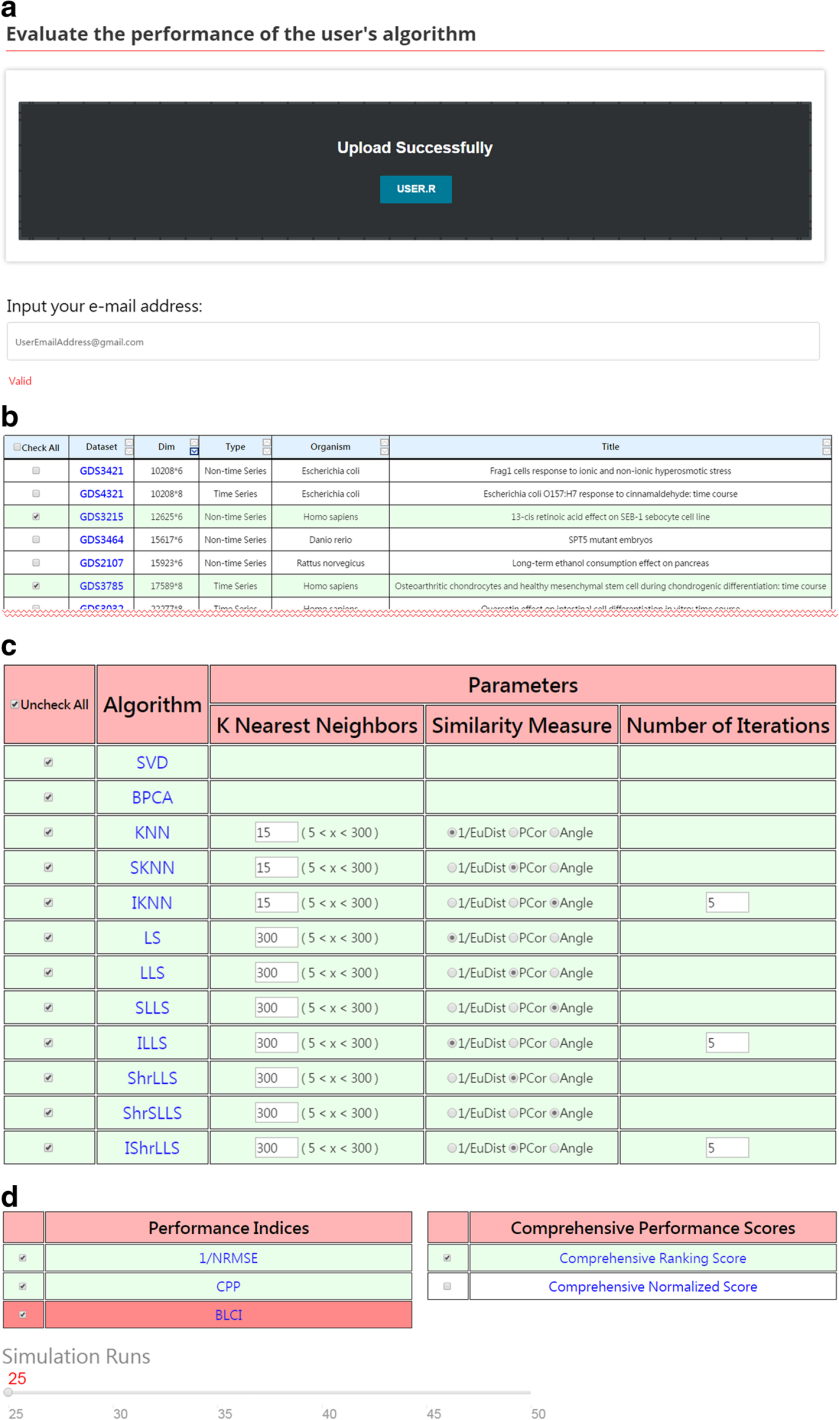
The input and five settings of MVIAeval. Users need to **a** upload the R code of their new algorithm, **b** select the test datasets among 20 benchmark microarray (time series or non-time series) datasets, **c** select the compared algorithms among 12 existing algorithms, **d** select the performance indices from three existing ones, the comprehensive performance scores from two possible choices, and the number of simulation runs

**Fig. 5:**
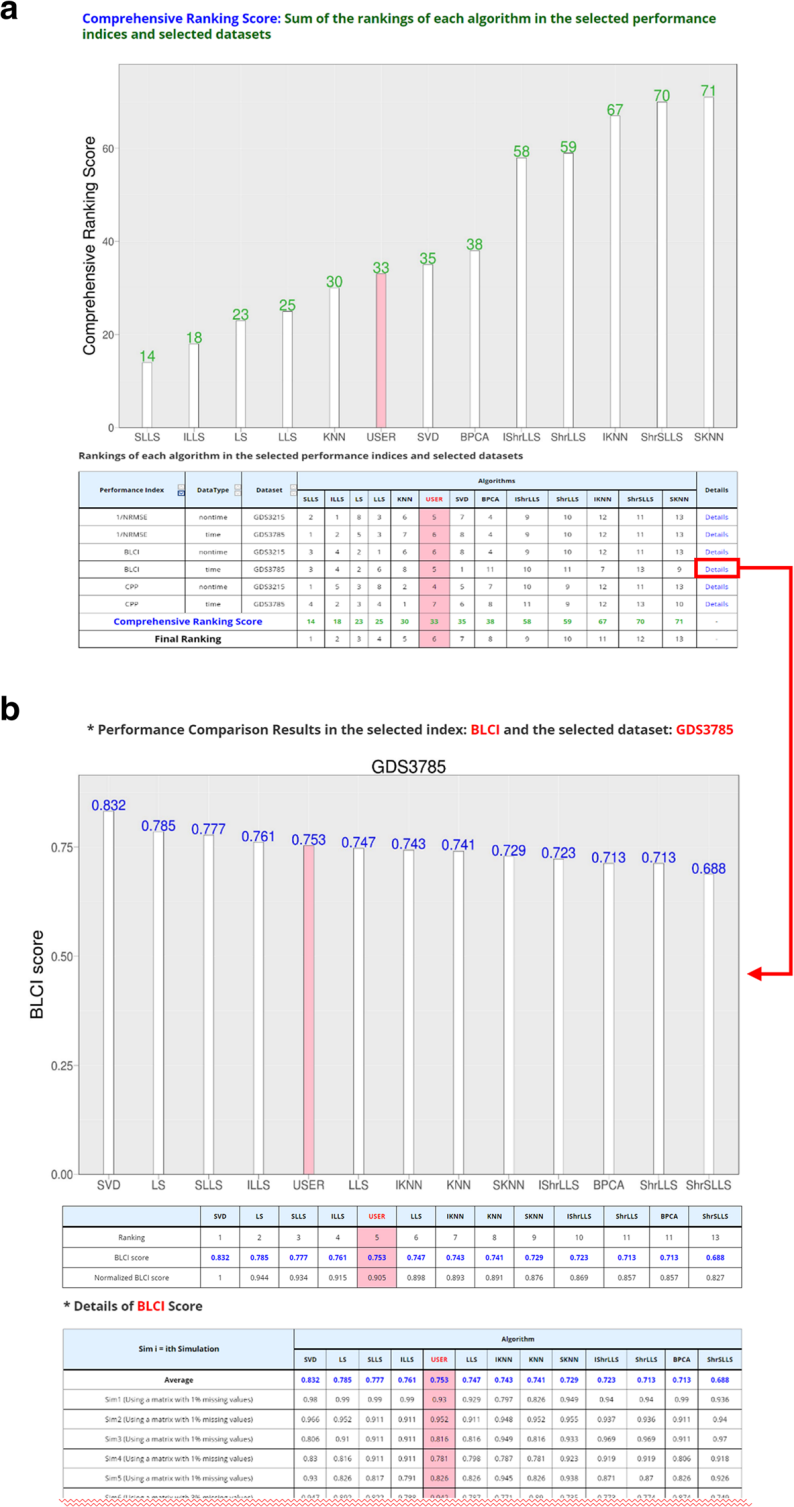
The output of MVIAeval. For demonstration purpose, we upload the R code of a sample algorithm as the user’s new algorithm and select two benchmark datasets (GDS3215 and GDS3785), 12 existing algorithms, three performance indices, the overall ranking score as the comprehensive performance score, and 25 simulation runs. **a** The webpage of the comprehensive performance comparison results shows that the overall performance of the user’s algorithm (denoted as USER) ranks six among all the 13 compared algorithms. **b** By clicking “details” in the row of BLCI for the benchmark dataset GDS3785, users can see the performance comparison results using only BLCI score for the benchmark dataset GDS3785. It can be seen that the user’s algorithm ranks five among the 13 compared algorithms using only BLCI score for the benchmark dataset GDS3785. The details of BLCI score for each algorithm can also be found

In MVIAeval, the R code of a sample algorithm is provided. For demonstration purpose, we regard the sample algorithm as the user’s newly developed algorithm and would like to use MVIAeval to conduct a comprehensive performance comparison of this new algorithm (denoted as USER) to various existing algorithms. For example, users may upload the R code of the new algorithm and select (i) two benchmark datasets, (ii) 12 existing algorithms, (iii) three performance indices, (iv) the overall ranking score as the comprehensive performance score, and (v) 25 simulation runs (see Fig. 4). After submission, MVIAeval outputs the comprehensive comparison results in both tables and figures. Among the 13 compared algorithms, the overall performance of the new algorithm ranks six (see Fig. 5). Actually, MVIAeval can provide the performance comparison results in many scenarios (see Table 3). It can be concluded that the new algorithm is mediocre because its performance is always in the middle of all the 13 compared algorithms in different data types (time series or non-time series), different performance indices (1/NRMSE, BLCI or CPP) and different comprehensive performance scores (ORS or ONS). Receiving the comprehensive comparison results from MVIAeval, researchers immediately know that there is much room to improve the performance of their new algorithm.

**Table 3:** MVIAeval can provide the performance comparison results in many scenarios

Performance Index	Benchmark datasets	Ranking of USER using ORS	Ranking of USER using ONS
1/NRMSE	Five Time Series [37–41]	5	6
	Five Non-time Series [27–31]	6	6
CPP	Five Time Series [37–41]	7	9
	Five Non-time Series [27–31]	11	8
BLCI	Five Time Series [37–41]	3	4
	Five Non-time Series [27–31]	7	7
1/NRMSE + CPP + BLCI	Five Time Series [37–41]	6	7
	Five Non-time Series [27–31]	6	6

The performance comparison results of the user’s algorithm (denoted as USER) and various existing algorithms using different types of datasets (time series or non-time series), different performance indices (1/NRMSE, CPP or BLCI), and different overall performance scores (overall ranking score (ORS) or overall normalized score (ONS)) are shown. More details could be seen at http://cosbi.ee.ncku.edu.tw/MVIAeval/A_Case_Study

## Conclusions

Missing value imputation is an inevitable pre-processing step of microarray data analyses. This is why the computational imputation of the missing values in microarray data has become a hot research topic. The newest algorithm is published in year 2016 [50] and we believe that many new algorithms will be developed in the near future. Using MVIAeval, bioinformaticians can easily get a comprehensive and objective performance comparison results of their new algorithm. Therefore, bioinformaticians now can focus on developing new algorithms instead of putting a lot of efforts for conducting a comprehensive and objective performance evaluation of their new algorithm. In conclusion, MVIAeval will definitely be a very useful tool for developing missing value imputation algorithms.

## Abbreviations

MVIAeval: Missing value imputation algorithm evaluator
NRMSE: Normalized root mean square error
CPP: Cluster pair proportion
BLCI: Biomarker list concordance index
ORS: Overall ranking score
ONS: Overall normalized score
